# Dermal Regeneration Template: Reconstruction in Oral Cancer Defects

**DOI:** 10.1007/s12663-023-01889-5

**Published:** 2023-03-21

**Authors:** Nicolò Mangini, Francesca Galvano, Resi Pucci, Andrea Battisti, Andrea Cassoni, Valentino Valentini

**Affiliations:** 1 Department of Oral and Maxillofacial Sciences, Sapienza University of Rome, Via Caserta 6, 00161 Rome, Italy; 2 Oncological and Reconstructive Maxillofacial Surgery Unit, Policlinico Umberto I, Viale del Policlinico 155, 00161 Rome, Italy

**Keywords:** Oral cancer, Dermal regeneration template, Integra, Mucosal reconstruction, Reconstructive surgery, Mucosal defects

## Abstract

**Background:**

Post ablative oral mucosal defect resulting from the removal of tumors can be treated with various techniques.

**Purpose:**

In this paper, we are showing what, in our experience, are the advantages and disadvantages given using biosynthetic skin substitutes when dealing with this kind of lesions.

**Materials and methods:**

Patients included in the sample came to our attention with both neoplastic lesions (11 subjects) and important scar retraction after previous oncologic surgery (1 subject). All patients underwent trans-oral resection surgery following the same surgical protocol and post ablative oral mucosal defect were treated using the dermal regeneration template. The surgical defect location, size, and time of removal of the silicone layer varied from one subject to the other.

**Results:**

Most patients showed good healing with reduced scarring and adequate remucosalisation of the defect. The main complications were shown in a palatal lesion treated with concomitant osteal resection, which developed an oroantral fistula at follow up, and tongue lesions which showed some scarring.

**Conclusions:**

Given our experience, we would advise using dermal substitutes when reconstructing oral defects only after a cautious evaluation of the area of the lesion, the gap size, the possible adherence of the membrane to the gap, and the presence of tissue supporting the overlying membrane.

## Introduction

When treating patients with oral cancer, it is crucial to consider an accurate tumor resection facilitated by the intraoperative assessment of the tumor-free margin and plan an adequate strategy for reconstruction. Often the post-ablative defect is not small enough to be closed for first intention, given the small amount of usable tissue in the mouth. Moreover, second-intention healing in the oral cavity could result in scarring and consequent contractures with functional defects [1]. Patients undergoing demolitive surgery for oral cancer treatment and free flap reconstruction might require surgery in the years following the first surgical treatment to obtain a neo-fornix and reduce scar retraction [2]. Those patients might also require autologous or heterologous grafts to fill the post-ablative defect. When the wound is wide, reconstructive flaps must be considered, evaluating both local flaps such as Bozola flap [3], FAMM flap [4] or buccal fat pad flap [5] and free flaps for reconstruction. Other strategies could be used in the in-between spectrum of post-ablative defect dimensions, including autologous grafts and dermal regeneration templates. At the moment, for intraoral defects not requiring regional or free flap for closure, it is possible to use autologous full-thickness or split-thickness grafts. The harvesting procedure of those grafts is not free of risks, including donor site infections, functional or aesthetic deficits, and patient discomfort [1]. The use of biosynthetic skin substitutes is increasing to avoid the risks above. Artificial dermal substitutes (DSs) help with physiological wound healing, ensuring consistent and enduring wound closure and providing a suitable scaffold to repair tissue [6]. This treatment allows healing with a lower risk of scar contractures and overall deficits without the patient undergoing a strict surgical procedure. Our study aims to evaluate the validity of DSs in reconstructing surgical defects in the oral mucosa and whether a different outcome could be related to a different lesion location.

## Materials and Methods

### Patients

This observational study was conducted between April 2021 and January 2022. The investigation was performed in compliance with the Declaration of Helsinki and the Guidelines for Good Clinical Practice (Prot. n. 0,000,208, 07/02/2022). All participants provided written informed consent to undergo surgery and follow-up. The inclusion criteria were Caucasian patients of either sex, with early T1 stage of oral cancers, precancerous lesions, or revision of previous surgical cancer treatment, that were treated with the dermal regeneration template either during the primary treatment or on secondary revision surgery. None of the patients in this study underwent radiotherapy or chemotherapy after DS placed surgery. The only patient with a DS placed for neo-fornix creation had previously undergone RT. All patients were treated at the maxillofacial surgery department of the “Policlinico Umberto I” hospital in Rome for ten months. The following data were gathered for each patient: demographic data, lesion site (palate, tongue, alveolar crest, trigonous and cheek), histology, staging, surgery procedure, comorbidities, healing time and complications.

### Surgical Protocol

**Fig. 1:**
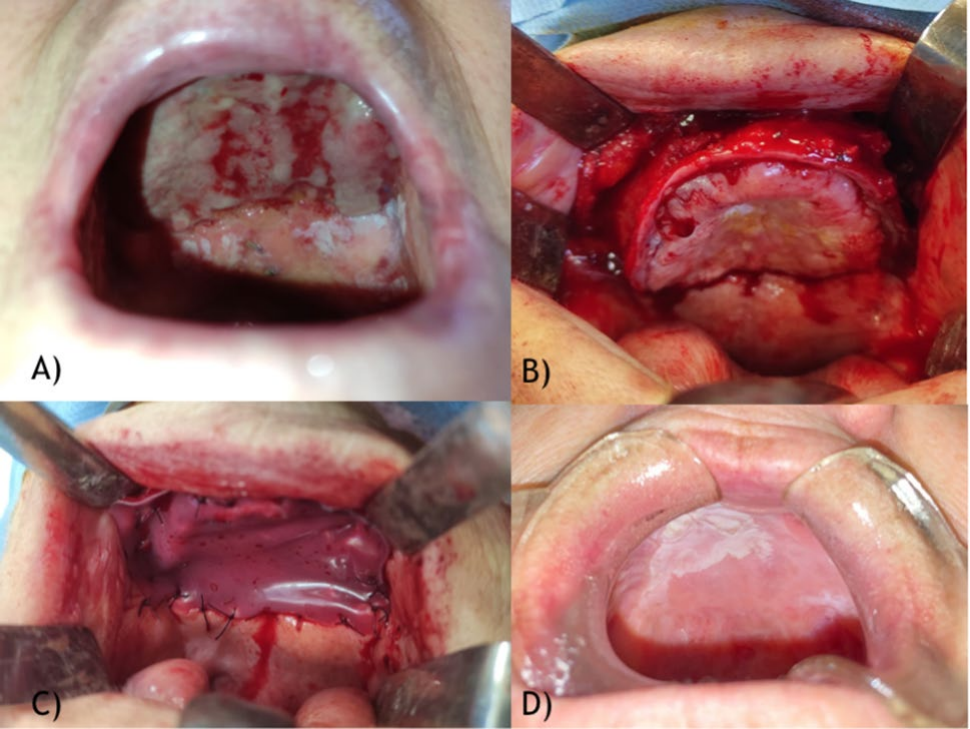
Palatal lesion. **A** Preoperative image; **B** Intraoperative image; **C** Integra® placement; **D** 9 months follow-up image

All patients with malignant lesions in need of primary surgery whose tumor did not exceed an early T1 cancerous stage (AJCC 8th Edition 2017) were previously discussed at the tumor board, where the indication for transoral resection was given; all patients underwent a transoral resection of the neoformation in wide free margins, performing intraoperative frozen sections of the margins. All tongue cancers underwent partial glossectomy in order to obtain a full-thickness excision of the lesion; cheek cancers underwent total thickness excision to the muscular plane, which was preserved with the intraoperative frozen section of the deep plane of resection resulting negative for neoplasm infiltration; retromolar trigon and alveolar crest lesions were treated via complete thickness excision including the periosteum. As for palatal cancers, one underwent the same treatment as the retromolar trigon lesions, while the other underwent a Brown I maxillectomy due to signs of bone infiltration; the same patient had previously undergone a Brown IIB right maxillectomy to treat a right superior alveolar crest G2 squamous cell carcinoma. Subsequent reconstruction of the gap using a 5 × 5 cm dermal substitute membrane from Integra® (Fig. 1) followed the demolitive surgery for all patients above**.** Patients who had previously undergone oral cancer treatment and came back to our department to solve the scar retraction from previous surgery were also included in this study; the neofornix was created using a 5 × 5 cm dermal substitute membrane from Integra® in order not to form new adherences and to make the fornix heal properly. The membrane shape and size were customized each time according to the gap using a template to adequately fit without excess or a tent-like effect. The layer was then accurately sutured in place using a Vicryl 3.0 suture. A compressive medication consisting of paraffin gauze anchored to the surrounding mucosa with a Silk 2.0 was used not to elevate the membrane from the gap. To ensure nutritional support, avoid contact between food and the membrane, and keep the site as clean as possible, enteral nutrition, managed by placing a nasogastric tube (NGT) at the end of the operative session, was set up in all but two cases, due to the impossibility for the patients to tolerate the NGT. In this case, a liquid diet was prescribed. Seriated medications were planned twice a week to check on the site and, when necessary, replace the gauze. The silicone layer of the membrane was removed between the 13th and 21st days post-surgery. In order to better evaluate the results, the patients were divided into groups based on the lesion location, and the outcomes were analyzed accordingly to highlight differences that might be related to the site, its mobility, and the ease of keeping the compressive medication in position.

### Integra® Bilayer Wound Matrix

Integra® Bilayer Matrix Wound Dressing (Integra LifeSciences, Princeton, NJ, USA) is a manufactured acellular dermal regeneration template made of a bilaminate sheet of cross-linked bovine tendon collagen and shark glycosaminoglycans (chondroitin-6-sulfate) with a silicone sheet cover [7]. Integra® acts in wound healing by stimulating natural recovery processes promoting localized inflammation, infiltration of neutrophils, macrophages, fibroblasts, and keratinocytes and neovascularization of the scaffold [6]. Integra® was initially created out of necessity to provide temporary coverage for patients with extensive burns. Those who benefited most from this technology were patients with severe full-thickness burns in whom donor sites were severely limited or nonexistent. However, since its first applications, the use of this biosynthetic substitute has widely increased. In particular, Integra® bilayer wound matrix is very effective in intra-oral reconstructions [1]. Its inner porous layer works as a scaffold guiding cellular migration and capillary invasion; the migration of blood vessels and other cells into the matrix allows the formation of a new layer of the dermis, while the outer silicone layer has a role in covering the wound surface, controlling moisture loss, and increasing tear strength of the matrix. Studies showed that the first layer is usually replaced within 14 to 21 days while the second, non-absorbable, is removed to allow epithelial growth [7]. When removing the silicone layer, remove the sutures and staples holding it in place, then with forceps and a spatula or other blunt instrument, lift the layer starting from the edge and gently peel back. After the removal of the silicone layer, eventually, a graft can also be added to the neodermis.

## Results

Eleven patients were included; 5 patients were males, and 7 females. The age of our patients ranged from 58 to 79 years old. Lesion locations included: tongue (5 patients), cheek mucosa (2 patients), palatal area (2 patients), alveolar crest (2 patients), and retromolar trigone (1 patient). Seven patients had malignant lesions, 6 squamocellular carcinoma (SCC) pT1 and 1 low-grade mucoepidermoid tumor pT1 [8], 2 pTIS [8], 1 had squamocellular papilloma, and 1 was treated for solving scarring from previous surgery for the resection of oral SCC and reconstruction with deep circumflex iliac artery (DCIA) free flap followed by radiation therapy. The most significant post-ablative defect covered with Integra was 3.8 × 4.3 cm. The average follow-up was 5 months. All the details of the sample are shown in Table 1.

**Table 1:** Characteristics of the sample

Location	Patient n°	Sex	Age	Treatment	Histology	TNM*	Previous treatment	Defect (cm)	Silicone removal	Complications	FU (mths)
Palate	1	F	75	Tumor removal including the periosteum	Verrucous carcinoma	pT1	none	4.3 × 3.8 cm	17th POD*	none	9
	2	F	69	Brown IA maxillectomy	SCC*	pTIS	Brown IIb right maxillectomy for previous right superior alveolar crest G2 SCC	3.7 × 3.2 cm	16th POD	oro-antral fistula (4 mm)	6
Retromolar Trigone	3	F	75	Tumor removal including the periosteum	Mucoepidermoid tumor	pT1	none	3.3 × 2.7 cm	15th POD	none	6
Tongue	4	M	69	Partial glossectomy	SCC	pT1	none	4.1 × 2.9 cm	5th POD	suspicion of infection	7
	5	M	70	Partial glossectomy	SCC	pT1	none	3.9 × 3.7 cm	13th POD	none	4
	6	M	65	Partial glossectomy	SCC	pT1	none	3.5 × 2.9 cm	14th POD	none	3
	7	M	63	Partial glossectomy	SCC	pT1	none	3.2 × 3.0 cm	15th POD	none	5
	8	M	65	Partial glossectomy	SCC	pTIS	Treated for SCC of the tongue in 2014: emiglosso-buccal pelvectomy + SND + reconstruction w/ ALT free flap2016: vestibular fornix deepening	3.7 × 3.1 cm	15th POD	none	4
Cheek mucosa	9	M	75	Tumor removal to the muscular plane	Squamocellular papilloma	/	none	3.5 × 3.0 cm	15th POD	none	4
	10	M	58	Tumor removal to the muscular plane	SCC	pT1	none	3.5 × 3.1 cm	15th POD	none	3

^*^TNM: 8th Edition TNM Classification^7^

*F* Female, *M* Male, *POD* Postoperative day

**Fig. 2:**
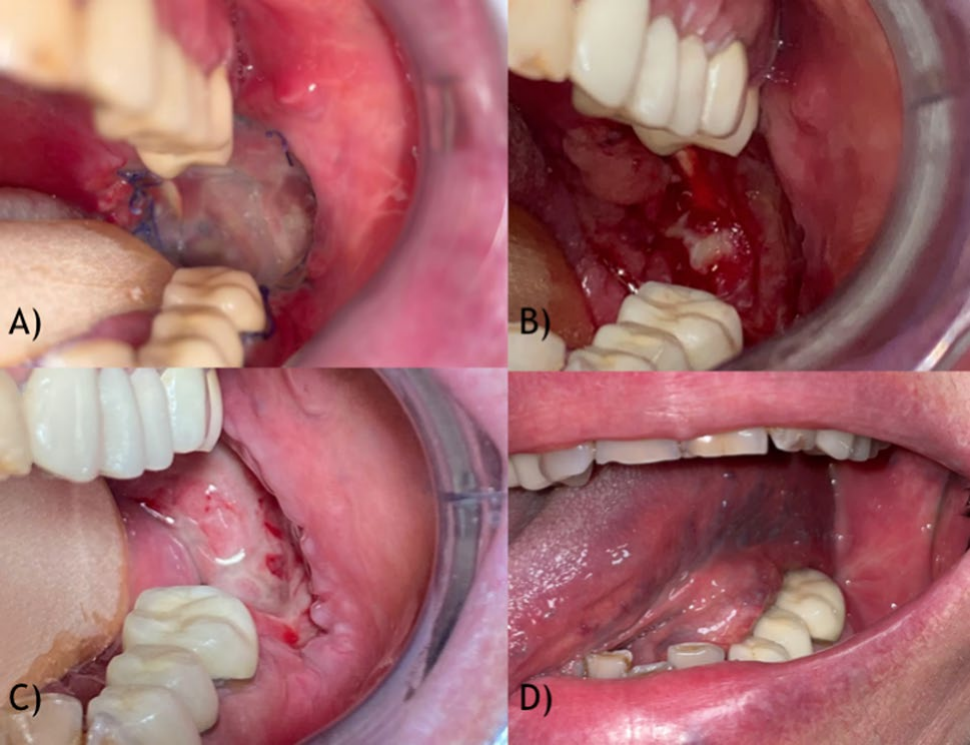
Retromolar trigone lesion. **A** 3 days postoperative image; **B** 20 days postoperative image with underlying granulating tissue; **C** 1-month postoperative image; **D** 6 months follow-up image

**Fig. 3:**
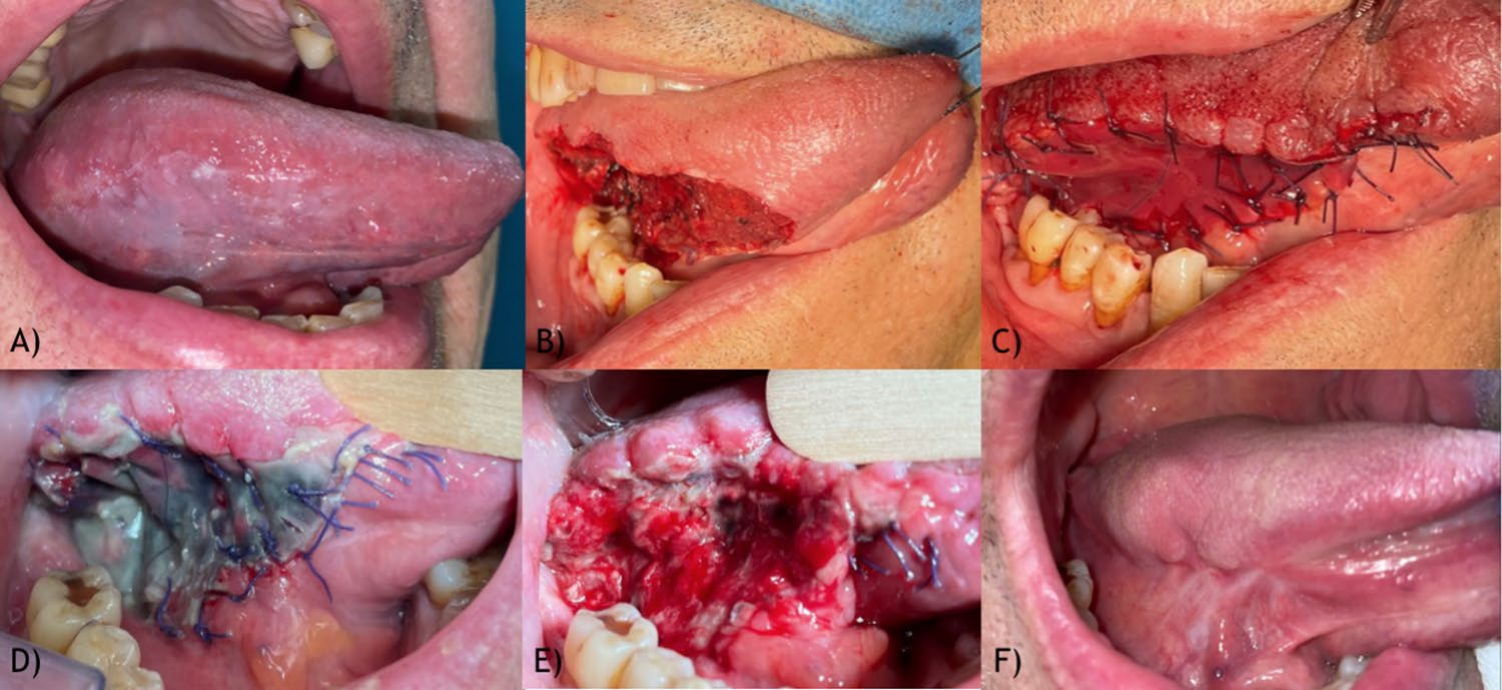
Tongue lesion. **A** Preoperative image; **B** Surgical gap; **C** Integra® placement; **D** 5th postoperative day, with suspects of infection, **E** 5th postoperative day, after silicone removal, healthy granulating tissue; **F** 7 months follow-up image

The 12 patients whose treatment was studied in this analysis underwent transoral surgery, all but one to treat oncologic lesions and one to treat scarring from previous tumor resection. All patients underwent surgery following the protocol stated above. As shown in Table 1, the silicone layer was removed on average on the 15th postoperative day showing in most patients a good amount of healthy granulation tissue underneath, as shown in Fig. 2. During clinical checkups, one patient with a tongue lesion had to have the silicone layer removed early (on the 5th day) due to a suspicion of infection (Fig. 3). A microbiological scrubbing was performed, which showed contamination by the normal bacterial flora of the oral cavity; the patient recovered without antibiotic therapy. The remaining patients with the same lesion area had the layer removed on the 13th day after surgery, showing a good amount of granulating tissue. After a 2-month follow-up, 2 patients showed good healing, while 3 showed some signs of scarring and retraction. Other complications were seen in a patient treated for a palatal lesion, who underwent partial palatal bone resection along with mucosal resection, and when the silicone layer was removed, an oro-antral fistula was shown. At the 2-month post-surgery follow-up, she presented a fistula measuring approximately 0.3 × 0.2 cm on the palate; therefore, a palatal obturator was conformed to allow proper alimentation and speech. After a 6-month follow-up, the fistula was completely healed. The last patient of the sample was operated on to treat scarring from previous cancer surgery. This patient had an oral prosthetic stent anticipatedly customized based on his teeth imprint, which was later used as a conformator to hold the paraffin gauze in place over the Integra matrix. In all the other patients treated through this protocol, healing proceeded smoothly as expected, and all completely healed at the 2-month postoperative mark.

## Discussion

The creation of dermal regeneration templates arises from the necessity to provide temporary covering to patients with extensive burns. The patients with severe full-thickness burns and donor sites severely limited or nonexistent benefited most from this technology. However, the clinical applications have broadened significantly since their initial applications in burn treatment. Integra® played a crucial role, widely studied, in the head and neck covering of full-thickness scalp wounds [9, 10]. It has also been utilized by Gravvanis et al. for intra-oral lining with permanent, durable covering. In this case, this DS was used, after free fibula flap reconstruction, to line the intraoral defect of the floor of the mouth over the neo mandible. It was also reported to have 100% take at removing the silicone layer after 2 weeks, with subsequent spontaneous mucosalization hard enough to support dental implants [11]. In our experience, the Bilayer Wound Matrix has always been used for wound closure on cutaneous gaps, with good overall results; therefore, since April 2021, we have started treating mucosal defects with the said dermal substitute.

Analyzing data collected from our patients, we can conclude that the results obtained using this kind of DS are strictly bounded to the surface where the membrane is placed; more specifically, the more the surface is mobile, the less tightly the membrane can adhere to it, allowing for less satisfactory results on more mobile surfaces, such as tongue defects. This can be explained because the inner layer of the membrane serves as a scaffold for cellular migration; the more this layer moves from the underlying surgical site, the more it does not allow a good recreation of the tissue beneath. Another obstacle to good mucosal regeneration we found was the absence of bone support in the palatal gap; our patient who underwent palatal bone resection and Integra® layer placement over the defect had great results on the areas where bone support was present underneath the membrane, but it was not able to suffice in closing the fistulas in the area where the bone had been resected. This could be explained by saying that the DS needs support from underneath, mucosa, periosteum, or bone. This is necessary for the migration of fibroblasts and endothelial cells that, attracted to the gap via the release of chemotactic factors, populate the scaffold given by the DS inner layer. During the study, we also highlighted that the patients on whom we fixed the membrane on the gap, not only with circumferential stitches but also with transfixed sutures through the membrane, had better outcomes. This is because the transfixed sutures allow a better adherence of the membrane to the surgical site also at the center of the lesion. As we can see, all the above can be related to the adherence of the membrane to the substrate underneath it.

Moreover, we noticed that, especially on mobile surfaces, there is a tendency for the bilayer to acquire a different coloring, making us think of infection and pushing towards an early removal of the silicone layer. When removing it, though, we noticed that the underlying defect was healing according to plan, and granulation was not affected; therefore, we concluded that infection must not be feared if no other signs other than membrane discoloration were present, and thus wait the usual 3 weeks to allow the inner layer to repopulate with cells and blood vessels. On our patients' sample, we noticed that the best timing for silicone layer removal was on the 21st day after surgery. For different reasons, we were not always able to keep it on for the suggested 3 weeks, but we saw that it was the best duration of the treatment without a higher risk of infection of the site. Moreover, we did not see a big difference in the healing process in patients with and without placement of an NGT if the patient was still able to have good oral hygiene. Therefore, in compliant patients, with good previous oral hygiene, it might not be necessary to place the NGT, informing the patient on the kind of diet to follow (semi-liquid) and the precautions to have when hygenising the oral cavity. Another limitation to using the dermal regeneration layer is its cost; being more expensive than most other closure techniques, it might not be usable on all patients in every setting. The limits of our study were the small sample of patients included, consisting of only 11 patients, not allowing us to have many cases for each lesion site to include in the study to draw adequate conclusions. Therefore, we recommend using the Integra layer for oral defect reconstruction, cautiously evaluating the gap site and the adherence of the membrane to the underlying surface.
